# Meeting report: a hard look at the state of enamel research

**DOI:** 10.1038/ijos.2017.40

**Published:** 2017-11-22

**Authors:** Ophir D Klein, Olivier Duverger, Wendy Shaw, Rodrigo S Lacruz, Derk Joester, Janet Moradian-Oldak, Megan K Pugach, J Timothy Wright, Sarah E Millar, Ashok B Kulkarni, John D Bartlett, Thomas GH Diekwisch, Pamela DenBesten, James P Simmer

**Affiliations:** 1 Program in Craniofacial Biology and Department of Orofacial Sciences, University of California, San Francisco, San Francisco, USA; 2 Department of Pediatrics and Institute for Human Genetics, University of California, San Francisco, San Francisco, USA; 3 Laboratory of Skin Biology, National Institute of Arthritis and Muscular and Skin Diseases, National Institutes of Health, Bethesda, USA; 4 Physical Science Division, Pacific Northwest National Laboratories, Richland, USA; 5 Department of Basic Science and Craniofacial Biology, New York University College of Dentistry, New York, USA; 6 Department of Materials Science and Engineering, Northwestern University, Evanston, USA; 7 Center for Craniofacial Molecular Biology, Herman Ostrow School of Dentistry, University of Southern California, Los Angeles, USA; 8 The Forsyth Institute, Cambridge, USA; 9 Department of Developmental Biology, Harvard School of Dental Medicine, Boston, USA; 10 Department of Pediatric Dentistry, University of North Caroline School of Dentistry, Chapel Hill, USA; 11 Departments of Dermatology and Cell & Developmental Biology, Perelman School of Medicine, and Department of Anatomy & Cell Biology, School of Dental Medicine, University of Pennsylvania, Philadelphia, USA; 12 Functional Genomics Section, National Institute of Dental and Craniofacial Research, Bethesda, USA; 13 Division of Biosciences, Ohio State University, College of Dentistry, Columbus, USA; 14 Department of Periodontics and Center for Craniofacial Research and Diagnosis, Texas A&M University College of Dentistry, Dallas, USA; 15 Center for Children’s Oral Health Research and Department of Orofacial Sciences, University of California, San Francisco, San Francisco, USA; 16 Biologic and Materials Sciences and Division of Prosthodontics, University of Michigan, Ann Arbor, USA

**Keywords:** enamel, mineralized tissue, mineralization, ameloblast, stem cell

## Abstract

The Encouraging Novel Amelogenesis Models and *Ex vivo* cell Lines (ENAMEL) Development workshop was held on 23 June 2017 at the Bethesda headquarters of the National Institute of Dental and Craniofacial Research (NIDCR). Discussion topics included model organisms, stem cells/cell lines, and tissues/3D cell culture/organoids. Scientists from a number of disciplines, representing institutions from across the United States, gathered to discuss advances in our understanding of enamel, as well as future directions for the field.

Enamel is a principal component of the dentition, and defects in this hard tissue are associated with a wide variety of diseases. To assess the state of the field of enamel research, the National Institute of Dental and Craniofacial Research (NIDCR) convened the “Encouraging Novel Amelogenesis Models and *Ex vivo* cell Lines (ENAMEL) Development” workshop at its Bethesda headquarters on 23 June 2017. Enamel formation involves complex developmental stages and cellular differentiation mechanisms that are summarized in Figure 1. The meeting, which was organized by Jason Wan from NIDCR, had three sessions: model organisms, stem cells/cell lines, and tissues/3D cell culture/organoids. In attendance were investigators interested in enamel from a broad range of disciplines as well as NIDCR leadership and staff. The meeting brought together developmental biologists, cell biologists, human geneticists, materials scientists, and clinical researchers from across the United States to discuss recent progress and future challenges in our understanding of the formation and function of enamel. Lively discussions took place throughout the day, and this meeting report highlights some of the major findings and ideas that emerged during the workshop.

## Model organisms

The meeting began with a discussion of the fundamentals of enamel formation by James Simmer (University of Michigan, Ann Arbor, MI, USA), who reviewed the stages of enamel development and discussed the conservation of the genetic and developmental aspects of enamel development during evolution. During the secretory stage, the enamel ribbons grow in length along the ameloblast distal membrane, which expands the enamel layer. During the maturation stage, the ribbons deposited during the secretory stage grow in width and thickness and the enamel layer hardens.

Enamel formation is a biological process that evolved in fish over 450 million years ago.^1-2^ The most distant surviving vertebrate (from humans) that makes enamel is the gar, which makes enamel on its scales (called ganoine) and also on its teeth. Studies of enamel formation in the gar,^3-4^ lungfish,^5^ mice and humans^6^ show that all enamel forms as characteristic thin mineral ribbons by a specialized mineralization front apparatus along the ameloblast distal membrane. Finger-like extensions of the ameloblast membrane initiate enamel ribbons on mineralized collagen fibers and elongate the ribbons as they retract back into the ameloblast membrane.^7^

Conserved events leading up to and including initial enamel formation are: (1) deposition of an unmineralized type I collagen matrix by the underlying mesenchymal cells (odontoblasts); (2) association of the tips of the collagen fibers with the epithelial (ameloblast) membrane, (3) fenestration of the basement membrane and the extension of finger-like ameloblast processes into the unmineralized collagen matrix; (4) the onset of dentin mineralization as discrete mineral foci; (5) expansion of the dentin mineral into a continuous layer; and (6) deposition of enamel ribbons on mineralized collagen by finger-like ameloblast membrane extensions that continue to extend the ribbons as they retreat into the ameloblast membrane.

**Figure 2:**
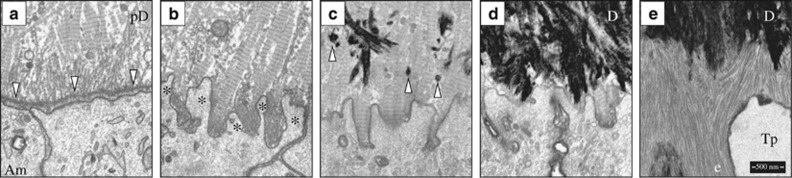
**Focused ion beam scanning electron microscopy (FIB-SEM) images of early dentin and enamel mineralization in the mouse mandibular incisor.** (**a**) The tips of unmineralized type I collagen predentin (pD) matrix deposited by odontoblasts (not shown) pass through the basement membrane (downward arrowheads) and associate with the ameloblast (Am) plasma membrane. (**b**) Fenestration of the basement membrane and the extension of ameloblast processes (*) into the unmineralized collagen matrix. (**c**) The onset of dentin mineralization as discrete mineral foci (upward arrowheads); (**d**) expansion of the dentin (**d**) mineral into a continuous layer; and (**e**) deposition of enamel mineral ribbons (**e**) on mineralized dentin. The ameloblast has already formed a Tomes process (Tp) that organizes the ribbons into rod and interrod enamel. FIB-SEM technology is allowing scientists to obtain ultrastructural information of enamel formation in wild-type and knockout mice.

Conservation is also observed at the genetic level. Early enamel formation in humans is dependent upon at least 4 secreted proteins, and inherited enamel malformations occur when the genes encoding them are defective: *ENAM*,^8^ *AMBN*,^9^ *AMELX*^10^ and *MMP20*.^11^ Enamelin and ameloblastin are specialized enamel proteins expressed during all vertebrate enamel formation and conserved in all species that produce enamel-like structures, including the ganoine of gar scales.^2^ No enamel ribbons form in *Enam* or *Ambn* knockout (KO) mice. Initial enamel ribbons form in amelogenin (*Amelx*) and matrix metalloproteinase 20 (*Mmp20*) null mice, but the process becomes progressively pathological over time. Dr Simmer also highlighted how the use of focused ion beam scanning electron microscopy (FIB-SEM) helped better understand the process of enamel ribbon formation along the ameloblast membrane^7^ (Figure 2).

Megan Pugach (Forsyth Institute, Cambridge, MA, USA) discussed mouse model approaches to enamel mineralization and pathology. One goal of enamel research is to regenerate enamel in humans, and animal models are required to study enamel formation due to their genetic tractability. Mouse and rat models with mutations in enamel genes that mimic *Amelogenesis Imperfecta (AI)* have proven essential for the study of complex enamel formation. However, these models have limitations including secondary effects, phenotype heterogeneity, random insertion of transgenes, and *Cre* recombinase toxicity. CRISPR/Cas9^12^ efficiently allows for multiplexed mutations to model genetic diversity of a disease such as caries or AI, to assess combinatorial gene effects, humanization of mice, and understanding of genetic backgrounds effects. Limitations of CRISPR/Cas9 include off-site effects and mosaicism.

While mouse models have enabled deep insights into enamel formation, it is difficult to directly translate results to the human system. This is due to the large differences in size, mineralization rate and anatomy. The non-primate genetically modifiable system closest to humans is the porcine, since they are diphyodont omnivores with molars, premolars, canines and incisors. Genetically modified porcine models can be used to study development and aging of teeth, enamel regeneration, function and mineralization kinetics, tissue engineering, oral microbiome interactions, metabolomics and transcriptomics. Because of the high cost of such models, consortia may be beneficial to enable advances with large mammalian models.

Rodrigo S Lacruz (New York University, New York, NY, USA) highlighted the important role of calcium (Ca^2+^) transport in enamel formation. Ca^2+^ ions are the most abundant in the composition of fully matured enamel, thus Ca^2+^ transport across enamel cells, and its accumulation in the extracellular space, is critically important for the formation and maturation of the enamel crystals. These functions are controlled by ameloblasts and dysregulation can have important detrimental effects in dental and oral health. Besides the critical role of Ca^2+^ in the structure of hydroxyapatite-like enamel crystals, Ca^2+^ is also an important intracellular second messenger with multiple cell functions.^13^ Changes in Ca^2+^ concentration in localized subcellular compartments mediate protein folding, generation of ATP, or regulate transcriptional networks.^14^ The effects of intracellular signals generated by changes in Ca^2+^ concentration depend on spatial and temporal characteristics, so that the entry point into the cells or its origin from the diverse intracellular Ca^2+^ stores generates a variety of signals.^15^ At any rate cells must monitor Ca^2+^ homeostasis as one of their principal functions and can do so by accumulating it in intracellular organelles or clearing it out of the cell. Thus, to fully understand the role of Ca^2+^ in enamel, it is important to monitor changes in Ca^2+^ concentration and movement within ameloblasts.

In healthy cells, mitochondria and endoplasmic reticulum (ER) store Ca^2+^ within their respective membranes. Cells use these intracellular stores to release Ca^2+^ into the cytosol as needed. However, the intracellular environment of the ameloblasts remains poorly understood hampering interpretations of how Ca^2+^ might modulate key functions of ameloblasts. An important contributor to Ca^2+^ entry is the store-operated Ca^2+^ entry (SOCE) pathway via the Ca^2+^ release activated Ca^2+^ (CRAC) channels mediated by STIM1 and ORAI proteins. Mutations in *STIM1* and *ORAI1* genes result in CRAC channelopathies (immune dysfunction, muscular hypotonia) including ectodermal dysplasia (severely hypomineralized enamel and anhidrosis), highlighting the dependence of enamel development on systemic dysfunction.^16^ The severity of the enamel malformations in patients with *STIM1* and *ORAI1* mutations is such that requires extensive dental restorations in both primary and permanent teeth.

Using *Stim1/2*-deficient mice to assess potential mechanisms associated with this dental abnormality, tight links between dysfunctions in Ca^2+^ homeostasis, ER stress and mitochondrial dysfunction that severely alter enamel mineralization were reported.^17^ It was also revealed recently that SOCE can modulate the expression of enamel genes *in vitro*, but data are limited. Although some progress is being made, much remains to be learned and understood about Ca^2+^ transport and signaling in enamel cells, the contribution of intracellular stores in this process, and how the intracellular environment of ameloblasts modulates the extracellular milieu and crystal growth.

The final talk in the first session was by Ashok Kulkarni (NIDCR, Bethesda, MD, USA), who examined challenges in developing genetically engineered mouse models to study enamel development and diseases. Many interesting mouse models are already available to study enamel biology, and strategies to make additional models to delineate precise role of candidate genes include: point mutations using CRISPR, cell autonomous role of specific genes using chimeras, generation of conditional nulls using cell type-specific *Cre* mouse lines, developmental stage-specific nulls using inducible *Cre*, humanized enamel mouse model, and somatic gene therapy using CRISPR and viral vectors. Various options can be pursued to select the proper model depending on the question(s) to be asked, and each one comes with certain limitations.

## Stem cells/Cell lines

The second session began with a talk by Ophir Klein (University of California, San Francisco, CA, USA) about regulation of epithelial stem cells in organ renewal. The continuously growing rodent incisor provides a model that allows us to understand how adult stem cells can produce progeny throughout an animal’s life.^18^ This system allows for powerful integration of investigations into how stem cells function, how they evolved, and how their behaviors are coordinated across tissues. This organ, like many others such as the skin, gastrointestinal tract, and hematopoietic system, is dependent on the continuous generation of progeny from stem cells that have the capacity to self-renew as well as to give rise to the required differentiated cell types. Candidate approaches to the identity and location of the stem cells are important,^19, 20, 21^ and unbiased screening techniques can be used to deconstruct the system.^22^ Several transcriptional and signaling networks that regulate the stem cells were discussed,^23, 24, 25, 26, 27, 28, 29^ and evolutionary perspectives on continuously growing teeth enabling cross-species comparisons^30-31^ were presented.

John Bartlett (Ohio State University, Columbus, OH, USA) surveyed ameloblast stress and enamel malformation. Soon after dentin mineralizes, enamel begins to form over the dentin outer surface on the developing unerupted tooth. During this time, an enamel organ covers the forming enamel and the cells in direct contact with this enamel are ameloblasts. Ameloblasts form a single columnar cell layer responsible for initiating enamel crystallite formation. As development progresses, approximately 10000 crystallites will coalesce into a single enamel rod^32^ and these rods span the entire length of the enamel layer starting from the dentin and finishing at the enamel surface. Ameloblasts are responsible for making the hardest substance in the body. Interestingly, ameloblasts are exquisitely sensitive to environmental stress. Nutritional deficiency, vitamin deficiency, congenital syphilis, hypocalcemia, birth injury, Rh hemolytic disease, local infection or trauma, ingestion of chemicals and febrile diseases such as measles, chicken pox and scarlet fever may cause enamel defects during enamel development.^33^ In fact, specific fields of paleontology are devoted to examining our ancestors’ teeth to determine not only what they may have eaten, but also to gauge their state of health.^34-35^ It remains unknown why ameloblasts, in particular, are so sensitive to stress.

Bartlett also discussed the hypothesis that moderate to high fluoride concentrations stress the ameloblasts responsible for enamel formation and that this stress causes the ameloblasts to become functionally compromised resulting in enamel dysplasia.^36^ Fluoride is a specific and effective caries prophylactic and its addition to drinking water at a concentration of 0.7 mg·L^−1^ is recommended by the centers for Disease Control and Prevention (CDC, 2011). Ingestion of fluoride at low concentrations hardens enamel making the enamel caries resistant. Nevertheless, moderate fluoride concentrations may cause white spot lesions on teeth and high fluoride concentrations may cause mottled, discolored enamel that is susceptible to decay.^37^

Tim Wright (University of North Carolina, Chapel Hill, NC, USA) presented on ameloblast gene expression and unraveling the human dentome. The formation of enamel involves a series of complex and highly controlled processes that are regulated by the expression of thousands of genes.^38^ Gene expression changes as the ameloblasts move through different developmental stages and related enamel forming processes. Not surprisingly, there are over one hundred conditions of known genetic origin affecting human enamel formation cataloged in OMIM (Online Mendelian Inheritance in Man). Most of the OMIM listed conditions with enamel phenotypes have a known associated or causative molecular defect with about 80% being associated with syndromes.^39^ Genes purported to be causative of these conditions code for extracellular matrix proteins, enzymes, transmembrane proteins, transcriptional factors, regulatory proteins and proteins with others functions. New hereditary conditions with enamel phenotypes continue to be cataloged and new genes associated with enamel defects are being identified.

To characterize the human transcriptome involved in enamel formation, human embryonic tooth buds were evaluated using a total genome RNA approach. The ameloblast transcriptome was interrogated at the presecretory and secretory development stages of development helping establishing a total developmental RNA profile of the tooth or the dentome. While most of the nearly 15 000 genes expressed by the ameloblast cells at the presecretory and secretory developmental stages of development were similar, there were a cluster of genes that showed differential expression.^40^ Some of the differences in gene expression were expected with up-regulation of enamel matrix associated genes such as *AMELX, AMBN* and *MMP20* in secretory ameloblasts compared with presecretory ameloblasts. Genes expressed by ameloblasts were diverse in their putative functions. Interestingly, genes showing expression in ameloblasts also are known to be expressed in other tissues such as renal and neural cells. It seems likely that some of these genes are critically important for normal enamel formation and will be implicated in hereditary defects of enamel in the future. In addition to the many genetic defects of enamel formation there are known to be a similar number of environmental etiologies (about 100) producing enamel phenotypes. Collectively, the environmental and genetic etiologies of enamel defects result in a high prevalence. Enamel hypoplasia is known to be a predisposing factor for the development of dental caries and conditions such as molar incisor hypomineralization contribute to the high morbidity of first permanent molars.

The third talk in this session explored stemness, lineage commitment and developmental potential in the enamel organ. To address the commitment and lineage differentiation potential of the four cell layers of the enamel organ,^41^ Tom Diekwisch and his team conducted studies to explain their fate and differentiation potential. The identity of the pre-ameloblast/ameloblast layer was defined by its proximity to the adjacent enamel mineral or dentin matrix, dependent on developmental stage. The stratum intermedium transiently expresses the epithelial stem cell marker p63, supportive of a potential role as an enamel organ stem cell layer that may provide a reservoir for ameloblast renewal. The stellate reticulum is linked to the papillary layer of the eruption stage tooth organ via keratin immunostaining. The papillary layer provides an important cell layer to facilitate infection-free tooth eruption. Finally, the outer enamel epithelium gives rise to the outer layer of Hertwig's Epithelial Root Sheath in mammals, while in reptiles, the outer enamel layer directly continues with the general lamina responsible for continuous successional tooth organ growth. Diekwisch therefore highlighted that the developing enamel organ is a multifunctional, complex cell assembly, in which different cell layers co-develop and synergize to serve various functions during enamel development, tooth organ succession, and tooth eruption.

The second session was closed by Sarah Millar (University of Pennsylvania, Philadelphia, PA, USA), who introduced her laboratory’s work focused on understanding cell-cell signaling mechanisms controlling development, stem cell function and regeneration of the epidermis and organs such as hair follicles, mammary glands, taste papillae and teeth that arise from embryonic ectoderm (ectodermal appendages). Her group has shown that Wnt/beta-catenin signaling is required for initiating the formation of hair follicles, mammary glands and taste papillae from multipotent cells in the embryonic surface ectoderm,^42, 43, 44^ and is necessary for multiple stages of tooth development.^45, 46^ Forced activation of this pathway promotes formation of ectopic teeth as well as other appendages, suggesting its potential utility in strategies for dental regeneration.^45, 47^ The Millar lab has also identified key roles for Wnt signaling in regulating the functions of adult epithelial stem cells.^48^ They showed that the Wnt ligand Wnt10a is required for normal levels of proliferation for a broad range of epithelial progenitor cells, including in hair follicles, epidermis, and taste and filiform papillae.^49^ In addition, Wnt10a/beta-catenin signaling promotes region-specific specialized differentiation programs in tongue filiform papillae and palmoplantar epidermis. Beta-catenin forms distinct transcriptional complexes in differentiating versus proliferating cells, enabling it to activate different sets of target genes.^49^ Millar emphasized that understanding the mechanisms through which signaling pathways such as Wnt/beta-catenin initiate and drive the development of ectodermal appendages such as the tooth is a crucial step towards regenerating dental tissues, including enamel.

## Tissues/3D cell culture/Organoids

The third session was opened by Pamela DenBesten (University of California, San Francisco, CA, USA), who explored how ameloblast differentiation requires stage specific, and yet to be identified, factors in the dental mesenchyme. Tooth loss due to genetic causes, trauma, caries or periodontal disease continues to be major health issue for both adults and children. Although dental implants have resulted in improved strategies to replace missing teeth, implants are a less optimal solution as compared to the ultimate goal of tooth regeneration. Key strategies for tooth regeneration were identified in the classic studies in mice conducted by Kollar and co-workers, who showed that either the early dental epithelium combined with non-dental mesenchyme, or the later differentiating bud-stage dental mesenchyme combined with non-dental epithelium, could result in tooth formation.^50, 51, 52, 53^ These mouse studies directed efforts for similar strategies that could be used for human tooth regeneration.

DenBesten *et al.*^54, 55^ found that when primary ameloblast lineage cells (ALCs) derived from either porcine or human developing tooth buds were cultured in 3D in matrigel, the cells formed acinar type structures that appeared similar to enamel pearls.^56^ They then determined that epithelial cells derived from human embryonic stem cells (hESCs) have characteristics similar to ALCs^57^ and used this cell source to explore the possibility that co-culture with human epithelial derived cells and dental pulp mesenchyme could result in tooth formation. They found that similar to the previously reported mouse studies, co-culture of the human epithelial cells with the human bud-stage dental mesenchyme resulted in the formation of tooth like structures. Co-culture of human epithelial cells with mature dental pulp from erupted human teeth formed acinar type epithelial structures, similar to those found when ameloblast lineage cells were cultured alone.^57-58^ These studies show the promise of human embryonic epithelial cells as a source for ameloblasts, and they raise the possibility that if adult dental pulp cells could be reprogramed to an earlier stage of differentiation, co-culture of these cells could regenerate teeth.

Derk Joester (Northwestern University, Evanston, IL, USA) presented a materials-centric view for assessment of model systems. From this perspective, good model systems recapitulate human enamel performance (for example, protection of dentin from wear over the lifetime of a human, formation of structurally similar caries lesions^59^), properties (such as wear resistance, toughness, and resistance to acid dissolution), hierarchical structure (including 3D weave of rods, arrangement of crystallites, presence of an amorphous intergranular phase) and ultimately the crystal growth process. While some data are available, a comprehensive view of how different model systems (murine, porcine, canine, bovine and human) differ in these interrelated areas is lacking, which makes comparisons difficult. In addition, there is also a lack of data on the heterogeneity of enamel within one tooth, between different teeth in the same individual, and between equivalent teeth in one species. While it is often held that continuously growing incisors are poor model systems for human teeth, Joester suggested that understanding developmental and functional differences between continuously erupting teeth and those that erupt just once remains an important topic of fundamental research.

Joester further discussed how imaging at the atomic scale, using atom probe tomography, reveals clues to amelogenesis and enamel function. Specifically, the existence of a relatively more soluble Mg-rich amorphous intergranular phase (AIGP) at the boundaries between enamel crystallites in rodent^60^ and human enamel^61^ appears to be linked to the susceptibility of enamel to biofilm-derived and ingested acids. Even within this AIGP, organics, carbonate and possibly water show distinct distribution patterns with important implications for the resistance to acid corrosion, mechanical properties, and the mechanism by which enamel crystals grow during amelogenesis.^62^ An important aspect of growth is the presence of magnesium during growth. At least in the rodent model, Mg is excluded from the growing crystal, which means that one would expect there to be an elevated concentration of it right at the interface between the mineral and the surrounding aqueous phase. The impact of Mg^2+^ on crystal growth and the interactions of enamel matrix proteins and peptides with the mineral and each other remain largely unexplored. The AIGP is likely dynamic in structure and composition, however, these dynamics are an underexplored field at this time. Finally, knowledge of the AIGP and its biogenesis may help engineer teeth that are chemically and or mechanically more robust. From an engineering point of view, the ability to accelerate tooth growth, and the impact of high growth rates on enamel defects will become of central importance in the future.

Janet Oldak (University of Southern California, Los Angeles, CA, USA) presented recent advances in understanding molecular interactions in the enamel matrix, and how these can move us towards the development of biomimetic strategies for synthetic enamel. Because dental enamel does not regenerate itself, efforts to develop improved biomaterials with mechanical and esthetic attributes close to those of natural enamel are timely and justified.^63-64^ To achieve the long-term goal of restoring dental enamel, it is necessary to understand the fundamental chemical and biological principles of extracellular matrix assembly and the manner they control mineral nucleation and growth.^65-66^ The key to achieving the precisely organized architecture of enamel lies not only in the activities of ameloblasts at the mineralization front, but also in the highly controlled expression of proteins and enzymes, and in the way these organic extracellular matrix (ECM) components (such as amelogenin, enamelin and ameloblastin) interact with each other, with the cells, and with the forming mineral.^67^ This dynamic mineralizing system offers scientists a wealth of information that allows for the study of basic principles of organic matrix-mediated biomineralization and can potentially be utilized in material science and engineering for development and design of biomimetic materials.^68^ In particular, enamel-inspired biomaterials could be developed as a future generation of dental restorative materials.

Wendy Shaw (Pacific Northwest National Laboratory, Richland, WA, USA) discussed interactions of enamel proteins with apatite (HAP). The dominant proteins in the developing enamel matrix are amelogenin, ameloblastin, amelotin and enamelin, and while all have a significant impact on the resulting enamel,^69^ amelogenin and splice variants with HAP are the most studied.^70, 71, 72, 73, 74, 75, 76, 77^ These studies demonstrate face-specificity to HAP, and specific residues from the protein, as well as a role for the single phosphoserine in altering phase transformation.^72, 73, 74, 76^ Common themes among enamel biomineralization proteins are that they are intrinsically disordered, they self-assemble (and likely assemble with each other), and it is likely that they interact with a variety of calcium phosphate phases. Relatively recent advances in atomic force microscopy (AFM) allow measurements of binding energies with a single face of HAP,^78^ as well as the structure of the protein bound to HAP and the orientation on HAP (using solid state NMR).^76, 79, 80, 81, 82, 83, 84, 85^ These measurements show slight changes in the energetics of protein-protein *vs*. protein-HAP interactions as a function of single site mutation, changes which could explain the alterations in enamel structure and morphology as a function of mutation. Overall it is a beautifully balanced system that is easily disrupted. Continued challenges include understanding structure-function relationships which is exacerbated by the intrinsically disordered nature and the prevalence to self-assemble, and understanding the timing and importance of different protein-protein interactions. Also a challenge is that quantitative structural and interfacial studies with atomic resolution can only be down *ex situ*, but over-simplifies the complexity of the developing enamel environment. Understanding the role of proteins in ion movement, and measuring transient structures of the proteins are also future challenges.

The final presentation of the day, by Olivier Duverger (NIAMS, Bethesda, MD), centered on the need for novel animal models for the study of tooth enamel in a tissue-specific manner. The most commonly used mouse line for conditional gene deletion in the enamel organ is the *K14-cre* line. The limitation of this line is that it deletes genes in all layers of the enamel organ: ameloblasts, stratum intermedium and papillary layer. In order to understand the cellular complexity of the organ that produces enamel, it would be essential to target gene deletion in subpopulations of cells in the enamel organ. The *AMELX-*cre line is readily available and can be used to target gene deletion specifically in ameloblasts from the secretory stage onwards.^86^ Specific deletion in the stratum intermedium has not been performed yet. However, the restricted expression of *Alpl* in the stratum intermedium^87^ gives potential for the development of *Alpl-creERT* mice that could be used for inducible deletion of genes in this compartment of the enamel organ. Characterizing the transcriptome of each layer of the enamel organ would help identify novel markers that are restricted to subcompartments of the enamel organ and develop genetic tools to dissect the function of each cell type in the process of enamel formation.

Duverger also discussed how enamel scientists can learn from other organs. Amelogenesis imperfecta is a rare monogenic disorder that can be found in non-syndromic and syndromic forms. The other organs affected in syndromic forms of *AI* include the skin, the kidney, the lungs, the eyes, the brain, and the immune system, among others. When studying genes involved in the pathogenicity of these forms of *AI*, understanding their function in the other affected organs can help elucidate their role in amelogenesis.

## Conclusions

The day ended with a group discussion focused on how to move the enamel field forward and more efficiently translate research findings to clinical applications. Even though tremendous progress has been made in the last century in our understanding of the genetic, physiologic and developmental mechanisms that drive the secretion and maturation of enamel, many crucial questions remain unanswered. What drives the formation of the Tomes’ process, the highly specialized compartment of ameloblasts that orchestrates enamel deposition? What makes ameloblasts migrate in a coordinated way that leads to enamel rod decussation? What is the involvement of the other cells that make up the enamel organ? What are the key determinants in the ion transport function of ameloblasts at each stage? Addressing these questions is an essential prerequisite to regenerating enamel in an *ex vivo* setting. Workshop participants were in general agreement that a major hurdle to progress is the need for heightened interaction among biologists and materials scientists. Enamel research is a multidisciplinary field, and one idea that was discussed was a platform where data and resources could be shared in a more efficient way. Participants also agreed that additional animal models would be useful to deepen our understanding of enamel formation *in vivo*. In addition, current ameloblast-like cell lines are limited in their replication cycles in culture, their ability to consistently generate typical enamel-like matrices, and their origin from different cell types of the enamel organ. Thus, the generation of improved ameloblast-like cell lines will enable scientists to exploit the ability of tissue-specific cells to form enamel-like extracellular matrices and facilitate faithful enamel crystal growth. Finally, the potential for organoid and organ on a chip technology in the future is quite exciting. Thus, there is enormous potential for progress in the field of enamel biology through increased interdisciplinary collaboration and improved approaches.

## Figures and Tables

**Figure 1:**
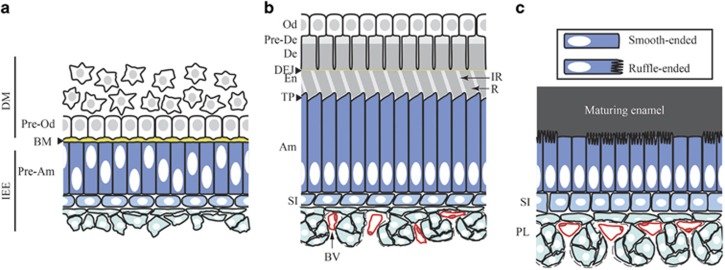
**Complex cellular interaction and differentiation processes involved in enamel development.** The pre-secretion (**a**), secretion (**b**) and maturation (**c**) stages of amelogenesis are represented in the context of the tissues surrounding the developing enamel. During tooth development, the formation of enamel and dentin, the two major mineralized constituents of the tooth, is initiated at the interface between the dental mesenchyme (DM) and the inner enamel epithelium (IEE), which are separated by a basement membrane (BM). The cells from the dental mesenchyme at this interface will differentiate into odontoblasts (Od) that produce predentin (Pre-De) and drive its progression into mineralized dentin (De). In the crown, cells from the inner enamel epithelium will differentiate into enamel-producing ameloblasts (Am). Prior to enamel and dentin deposition (pre-secretion stage), interactions between pre-odontoblasts (Pre-Od) and pre-ameloblasts (Pre-Am) play a crucial role in the specification of both compartment. Pre-dentin is secreted first and is comprised mainly of type I collagen, which starts to mineralize. Pre-ameloblasts secrete enamel matrix proteins and initiate enamel mineral ribbon deposition at the dentin-enamel junction (DEJ). Ameloblasts then go through a secretory stage where they deposit enamel matrix proteins into highly structured enamel rods (R) and interrods (IR). During this stage ameloblasts are elongated and develop a specialized structure at the secretion front called the Tomes’ process (TP). The secretion phase is followed by a maturation phase during which enamel matrix proteins are degraded by proteases to leave space for the full expansion of the hydroxyapatite crystals. During this stage, ameloblasts are shorter and cycle between ruffle-ended and smooth-ended phases. The epithelial cells underlying the ameloblasts progressively develop into a stratum intermedium (SI), directly in contact with the ameloblasts, and a papillary layer (PL) populated by blood vessels (BV). Although these layers certainly play an important role in enamel development, their function remains poorly understood.
